# Supplementary material for: Regulation of plasma triglyceride partitioning by adipose-derived ANGPTL4 in mice
Source: Sci Rep. 2021 Apr 12;11:7873. doi: 10.1038/s41598-021-87020-5 (PMC8041937; doi:10.1038/s41598-021-87020-5)
Supplement: Supplementary file 1 — Supplementary Information [file 41598_2021_87020_MOESM1_ESM.pdf]

Regulation of plasma triglyceride partitioning by adipose-derived ANGPTL4 in mice

Kathryn M. Spitler[1], Shwetha K. Shetty[1], Emily M. Cushing[1], Kelli L. Sylvers-Davie[1], and Brandon S.J. Davies[1]*

[1] Department of Biochemistry, Fraternal Order of Eagles Diabetes Research Center, and Obesity Research and Education Initiative, University of Iowa, Iowa City, IA 52242

*Address Correspondence to: Brandon S. J. Davies, Department of Biochemistry, University of Iowa, 169 Newton Rd., PBDB 3326, Iowa City, IA, 52242. Tel.: 319-335-3225; Fax: 319-335-9570; E-mail: Brandon-davies@uiowa.edu.

Supplemental Data

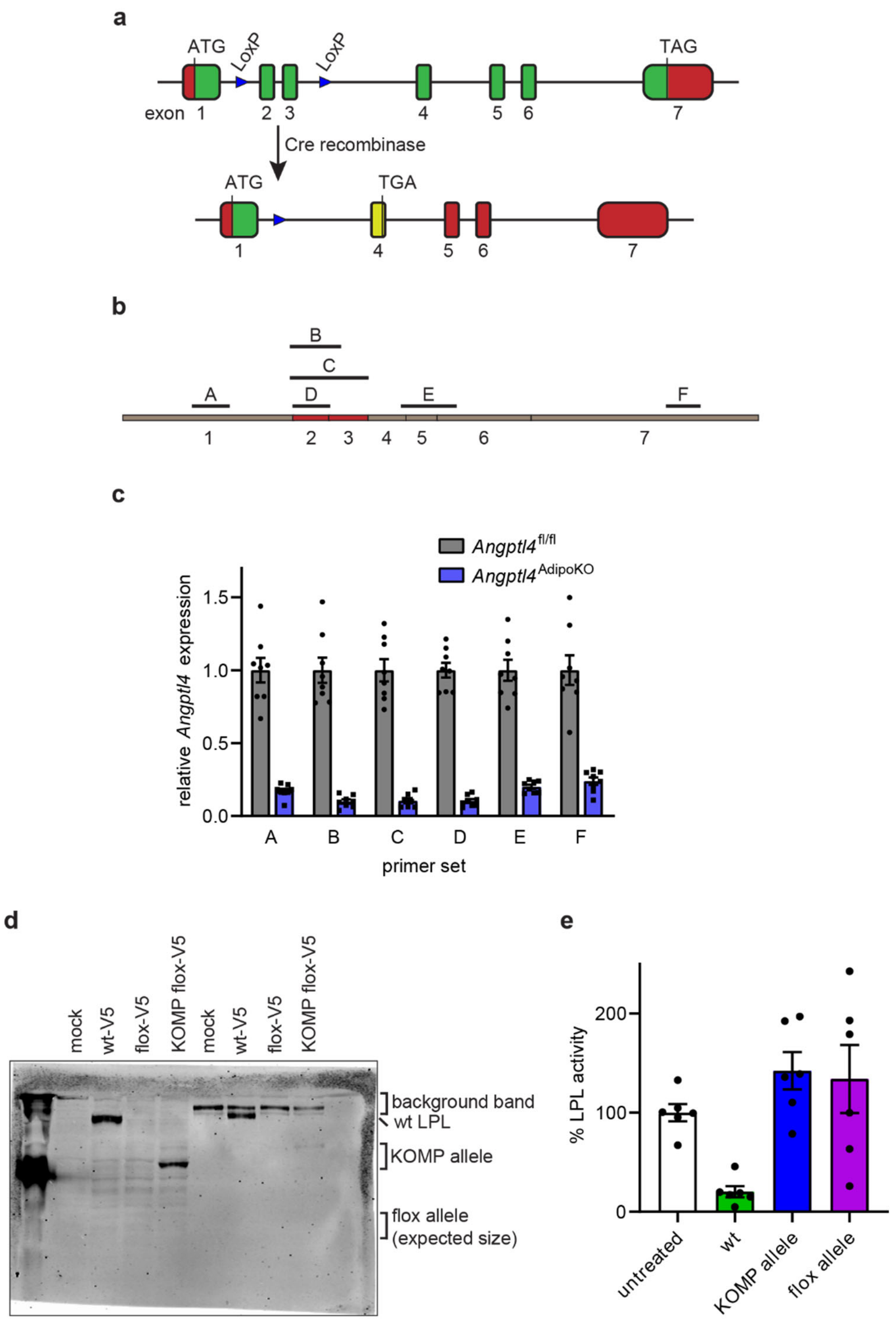

Supplemental Figure 1: Characterization of *Angptl4* flox allele. a) Schematic illustration of the lox-P sites in the *Angptl4*$^{fl/fl}$ allele and genomic rearrangement that results from Cre-mediated recombination. **b)** Schematic of the coding region of the ANGPTL4 gene showing the regions amplified by primers A–F. **c)** mRNA expression of *Angptl4* in brown adipose tissue of *Angptl4*$^{fl/fl}$ and *Angptl4*^{AdipoKO} mice using primers A–F (mean±SEM; n=7–8). **d)** Western blot of tissue lysate and conditioned media from 293T cells transfected with constructs encoding V5-tagged full length mouse ANGPTL4, V5-tagged Flox mouse ANGPTL4 or V5-tagged KOMP allele mouse ANGPTL4 probed with antibody against the V5 epitope. **e)** LPL activity of LPL treated with conditioned media from cells transfected with constructs encoding V5-tagged full length mouse ANGPTL4, V5-tagged flox mouse ANGPTL4, or V5-tagged KOMP allele mouse ANGPTL4 (means±SEM of three experiments with n=2 per group per experiment).

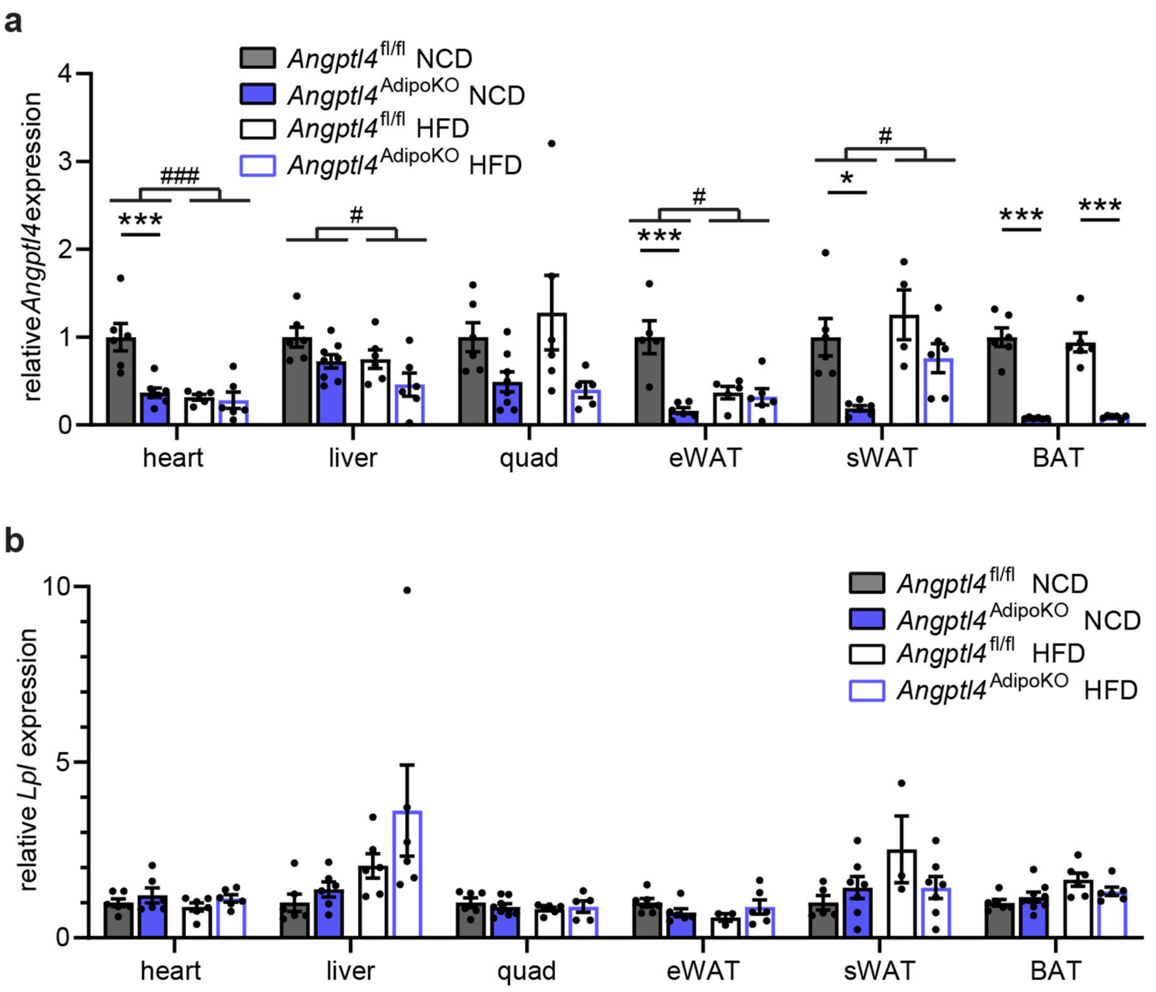

Supplemental Figure 2: *Angptl4* and *Lipoprotein lipase* expression in male *Angptl4*$^{fl/fl}$ and *Angptl4*^{AdipoKO} mice fed a NCD or HFD for 12 weeks. Fasted (6 h) mRNA expression of *Angptl4* **(a)** and *Lpl* **(b)** in liver, heart, quadriceps muscle (quad), epididymal white adipose tissue (eWAT), subcutaneous white adipose tissue (sWAT), and brown adipose tissues (BAT) of male *Angptl4*$^{fl/fl}$ and *Angptl4*^{AdipoKO} mice fed either a normal chow diet (NCD) or a high fat diet (HFD; 60% by kCal) for 12 weeks starting at 8 weeks of age (mean±SEM; n=3–8/group). #p<0.05, ##p<0.01, ###p<0.001 for dietary differences by two-way ANOVA. *p<0.05, **p<0.01, ***p<0.001 for individual genotype-specific differences by multiple comparison after two-way ANOVA (Tukey correction).

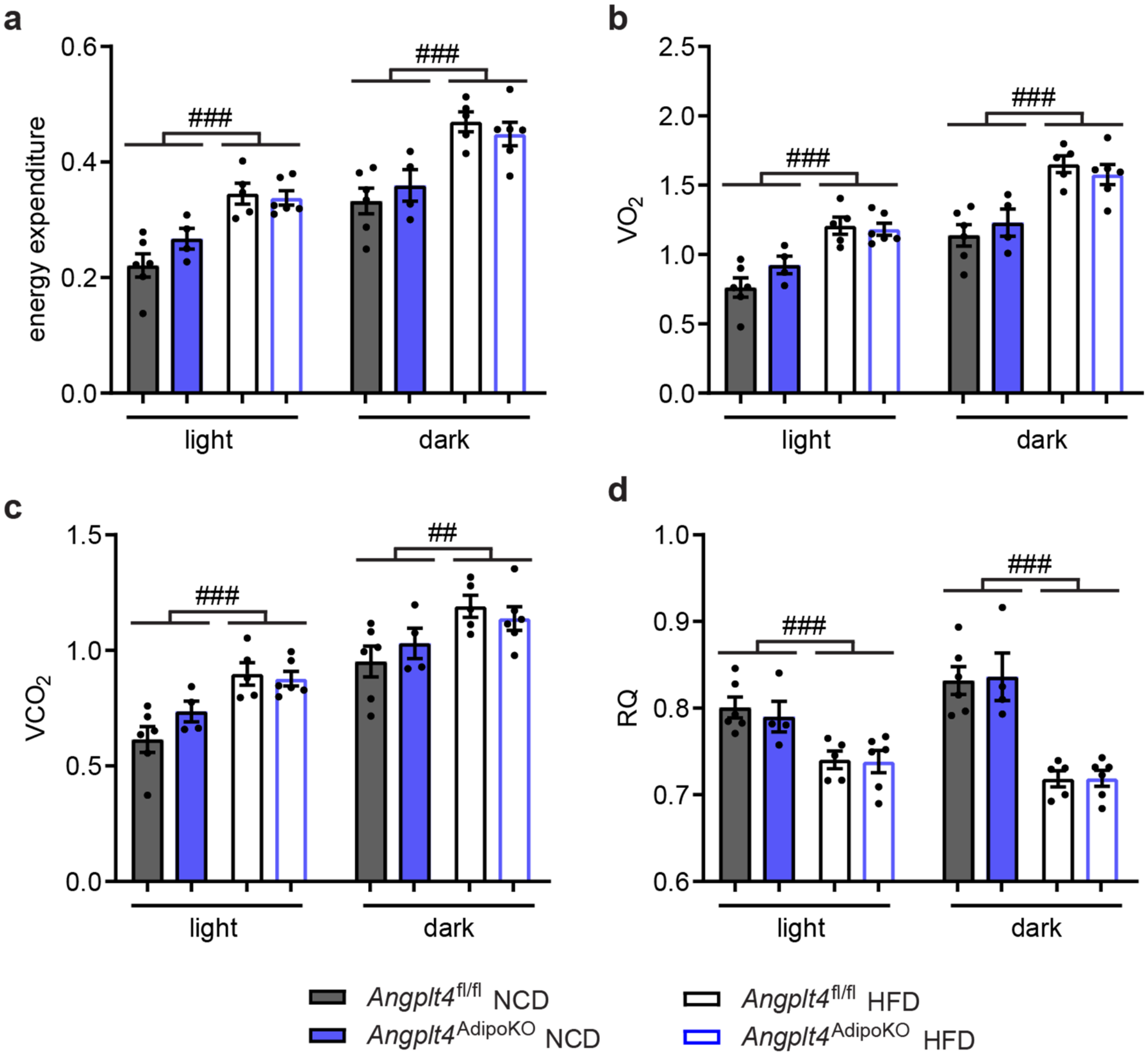

Supplemental Figure 3. Respiratory measurements in male *Angptl4*[fl/fl] and *Angptl4*[AdipoKO] mice fed a NCD or HFD for 12 weeks. After 12 weeks on NCD or HFD, male *Angptl4*[fl/fl] and *Angptl4*[AdipoKO] mice were placed into metabolic cages. After 36 h of acclimation, energy expenditure **(a)**, VO_2 **(b)**, VCO_2 **(c)**, and respiratory quotient **(**RQ**, G, H)** were measured over 48 h (mean±SEM; n=4–6). Data are separated into the light (6 a.m–6 p.m.) and dark (6 p.m.–6 a.m.) cycles. ##p<0.01, ###p<0.001 for dietary differences by two-way ANOVA.

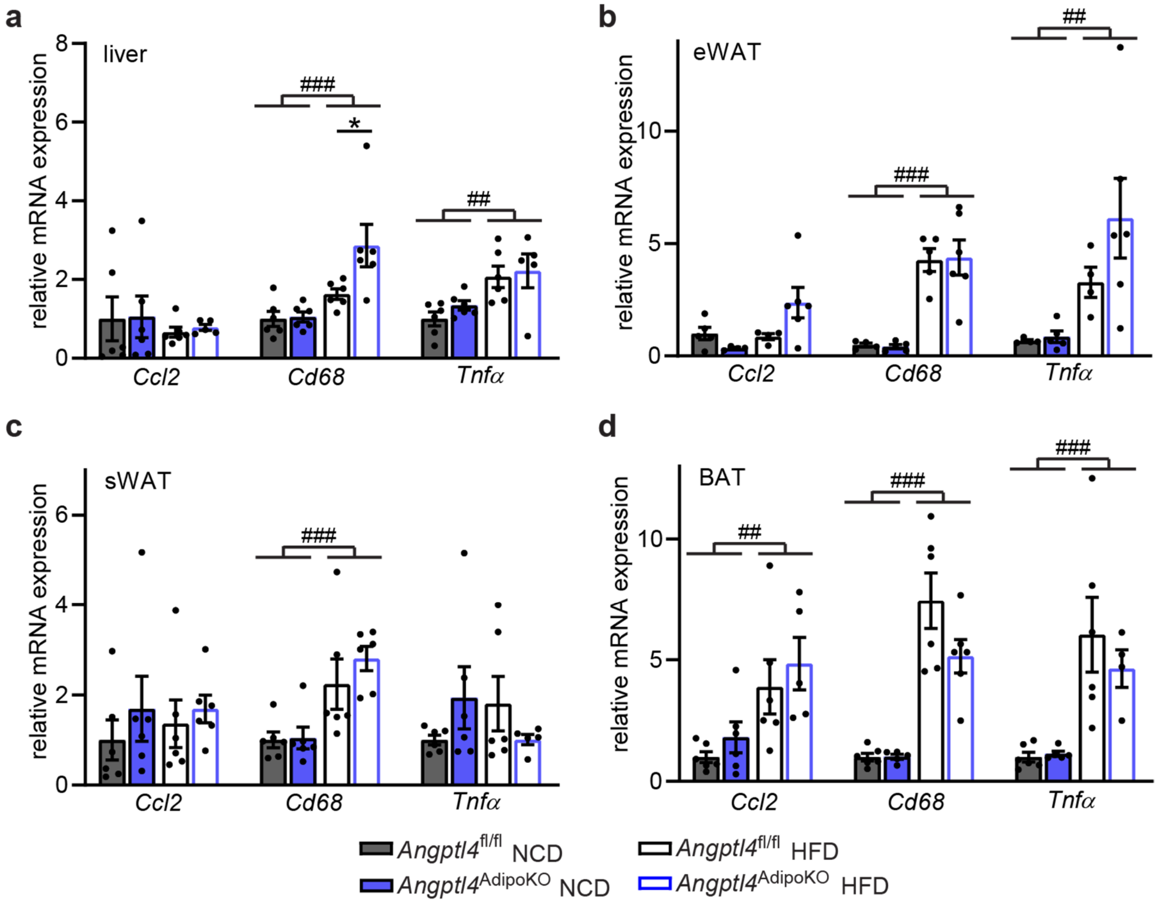

Supplemental Figure 4: Inflammatory marker expression in tissues from *Angptl4*$^{fl/fl}$ and *Angptl4*^{AdipoKO} mice fed a NCD or HFD for 12 weeks. Fasted (6 h) mRNA expression of inflammatory markers *Ccl2*, *Cd68*, and *Tnf*α from liver tissue **(a)**, gonadal white adipose tissue (eWAT) **(b)**, subcutaneous white adipose tissue (sWAT)(**c**), and brown adipose tissues (BAT)**(d)** of male *Angptl4*$^{fl/fl}$ and *Angptl4*^{AdipoKO} mice fed either a normal chow diet (NCD) or a high fat diet (HFD; 60% by kCal) for 12 weeks starting at 8 weeks of age (mean±SEM, n=6/group). ##p<0.01, ###p<0.001 for dietary differences by two-way ANOVA. *p<0.05, for individual genotype-specific differences by multiple comparison after two-way ANOVA (Tukey correction).

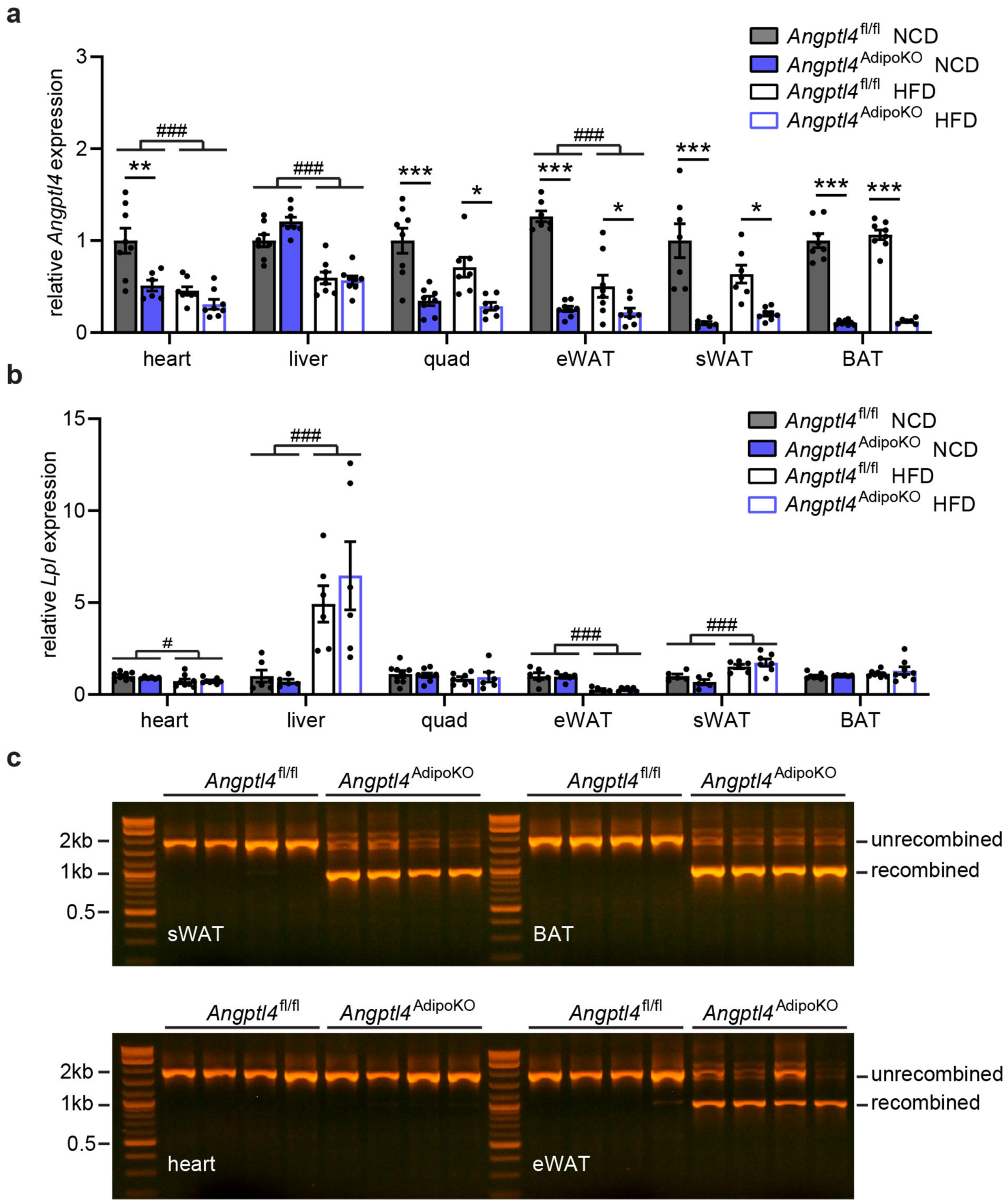

Supplemental Figure 5: *Angptl4* and *Lpl* expression in *Angptl4*^{AdipoKO} mice after chronic high-fat feeding. mRNA expression of *Angptl4* **(a)** and *Lpl* **(b)** from heart, liver, quadriceps muscle (quad), gonadal white adipose tissues (eWAT), subcutaneous white adipose tissue (sWAT), and brown adipose tissues (BAT) of fasted (6 h) male *Angptl4*$^{fl/fl}$ and *Angptl4*^{AdipoKO} mice after 6 months on NCD or HFD (mean± SEM; n=5–8/group). ##p<0.01, ###p<0.001 for dietary differences by two-way ANOVA. *p<0.05,**p<0.01,***p<0.001 for individual genotype-specific differences by multiple comparison after two-way ANOVA (Tukey correction). **c)** Agarose gel image of PCR amplification of *Angptl4* genomic DNA isolated from sWAT, BAT, eWAT and Heart of fasted (6 h) male *Angptl4*$^{fl/fl}$ and *Angptl4*^{AdipoKO} mice after 6 months on NCD (n=4/group).

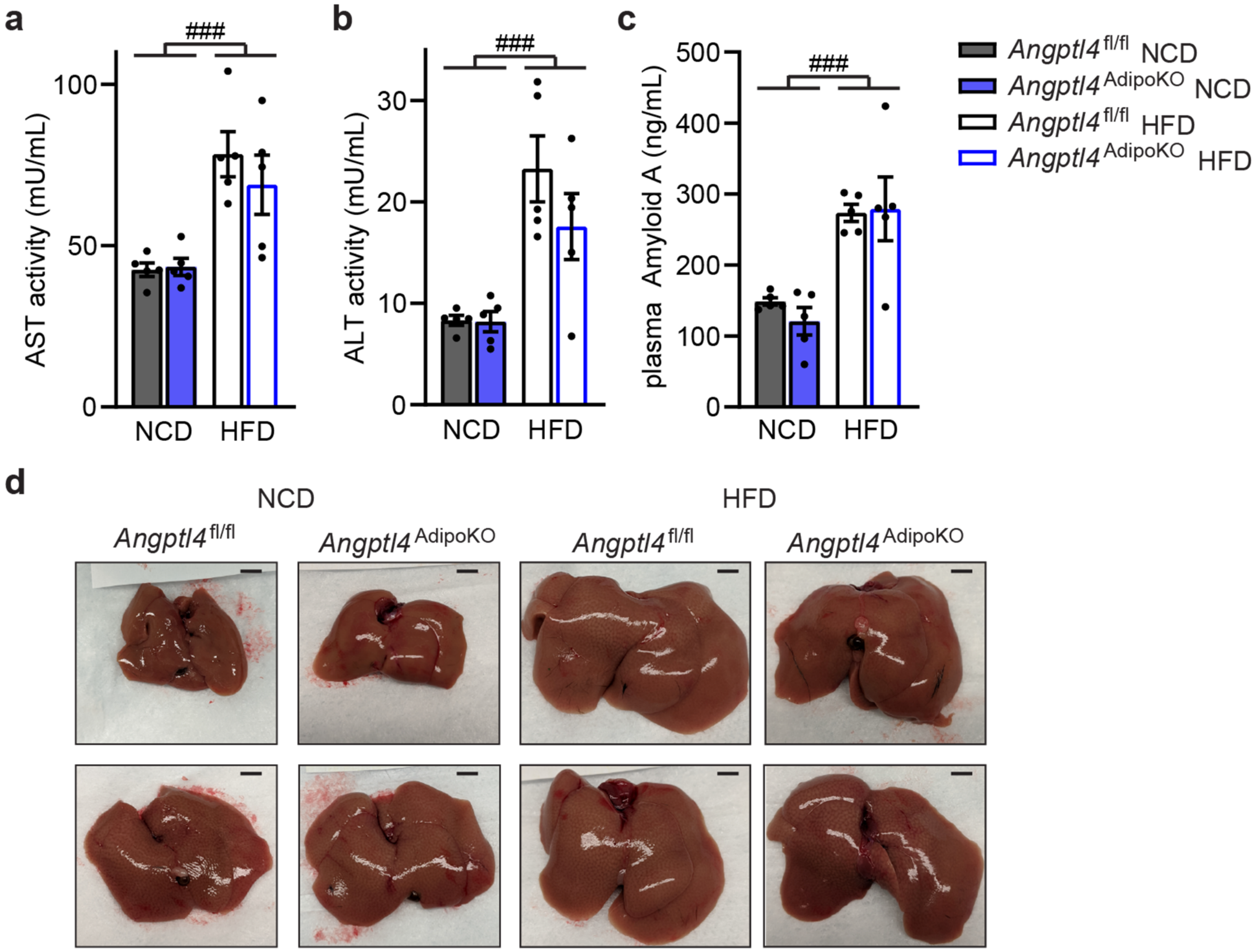

Supplemental Figure 6: Liver phenotypic markers in *Angptl4*$^{fl/fl}$ and *Angptl4*^{AdipoKO} mice after chronic high-fat feeding. Aspartate Aminotransferase (AST) activity **(a)**, Alanine Aminotransferase (ALT) activity **(b)** and Amyloid A levels **(c)** from the plasma of fasted (6 h) male *Angptl4*$^{fl/fl}$ and *Angptl4*^{AdipoKO} mice fed either a NCD or HFD for 6 months (mean±SEM, n=5/group). ###p<0.001 for dietary differences by two-way ANOVA. Representative pictures of livers from male *Angptl4*$^{fl/fl}$ and *Angptl4*^{AdipoKO} mice **(d)** after 6 months of either NCD or HFD feeding.

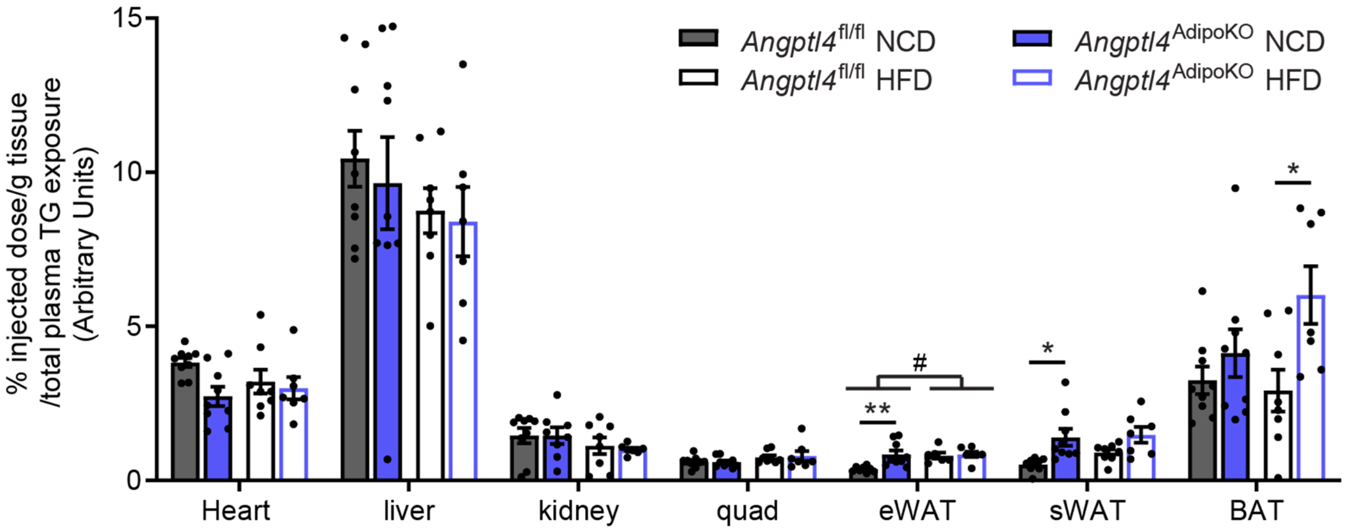

Supplemental Figure 7: Chylomicron TG uptake after chronic high-fat feeding normalized to plasma TG exposure. Each point represents the uptake of radiolabel (% injected dose/g tissue) into the indicated tissues after 15 min (as shown in figure 6b) divided by the area under curve of the plasma clearance (as shown in figure 6a) as calculated for each individual mouse. #p<0.05 for dietary differences by two-way ANOVA. *p<0.05, **p<0.01 for individual genotype-specific differences by multiple comparison after two-way ANOVA.

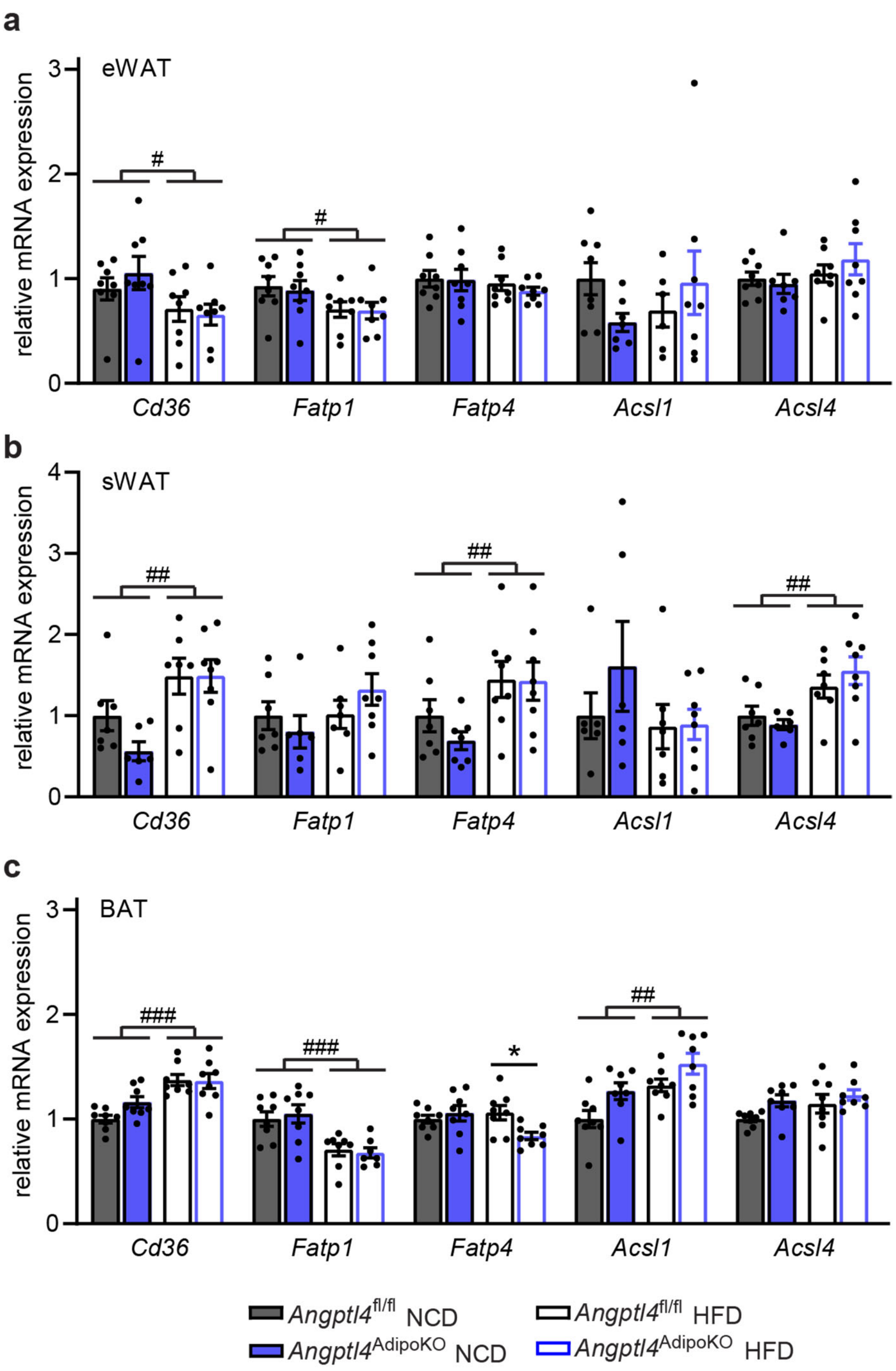

Supplemental Figure 8: Fatty acid transport and synthesis marker expression from *Angptl4*$^{fl/fl}$ and *Angptl4*^{AdipoKO} mice after chronic high-fat feeding. Fasted (6 h) mRNA expression of fatty acid transport markers *Cd36*, *Fatp1*, and *Fatp4* and fatty acid synthesis markers *Acsl1* and *Acsl4* from epidydimal white adipose tissue (eWAT)**(a)**, subcutaneous white adipose tissue (sWAT)**(b)**, and brown adipose tissues (BAT)**(c)** of male *Angptl4*$^{fl/fl}$ and *Angptl4*^{AdipoKO} mice fed either a normal chow diet (NCD) or a high fat diet (HFD) for 6 months (mean±SEM, n=6–8/group). #p<0.05, ##p<0.01, ###p<0.001 for dietary differences by two-way ANOVA (Tukey correction).

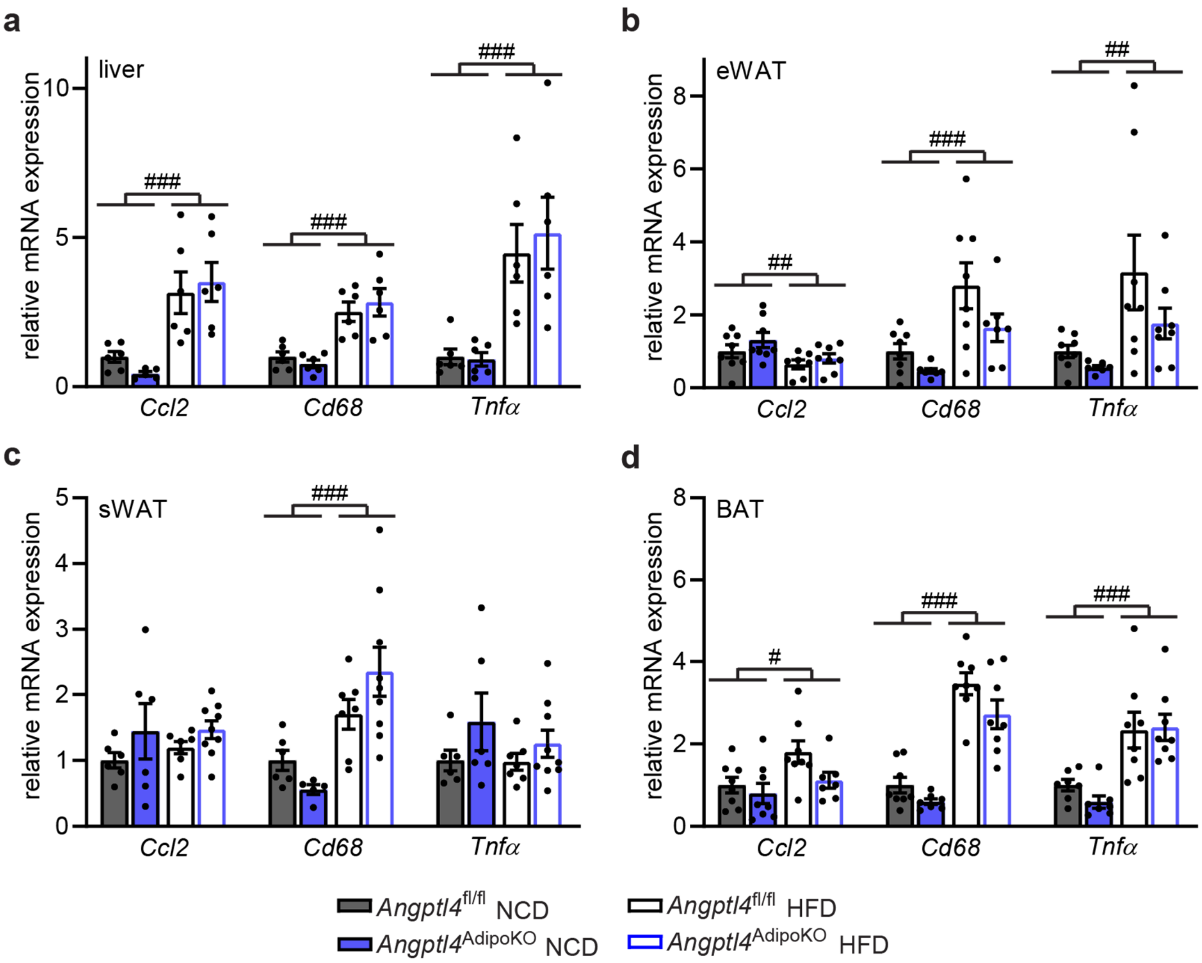

Supplemental Figure 9: Inflammatory marker expression from *Angptl4*fl/fl and *Angptl4*AdipoKO mice after chronic high-fat feeding. Fasted (6 h) mRNA expression of inflammatory markers *Ccl2*, *Cd68*, and *Tnf*α from liver tissue **(a)**, gonadal white adipose tissue (eWAT)**(b)**, subcutaneous white adipose tissue (sWAT)**(c)**, and brown adipose tissues (BAT)**(d)** of male *Angptl4*fl/fl and *Angptl4*AdipoKO mice fed either a normal chow diet (NCD) or a high fat diet (HFD; 60% by kCal) for 6 months (mean±SEM, n=6–9/group). #p<0.05, ##p<0.01, ###p<0.001 for dietary differences by two-way ANOVA.

Supplemental Table 1: Primers A–F used in Supplemental Figure 1

Primer	Forward	Reverse
**A**	GCTGCCCTGGTGCTATG	TGAGCCAGCAAGTTCATCTC
**B**	TTTGCAGACTCAGCTCAAGG	TCCATTGTCTAGGTGCGTGG
**C**	TTTGCAGACTCAGCTCAAGG	TGTGTAAGTGGGTGGCGTTGGG
**D**	GACTCAGCTCAAGGCTCAAA	TCTGGCTCTGAAGATTCTGTATTC
**E**	ATGACTTCAGATGGAGGCTGG	AATTGGCTTCCTCGGTTCCC
**F**	CAACTAGCTGGGCCCTTAAT	ATCCACAGCACCTACAACAG

Supplemental Table 2: Primers used in Supplemental Figures 2,4,5,8,9

Primer	Forward	Reverse
Angptl4	TTTGCAGACTCAGCTCAAGG	TCCATTGTCTAGGTGCGTGG
Lpl	CTGGTCTTAACCGGCCCAAT	TGCACATAGCCAGAAGGGTG
Ccl2	CCCAATGAGTAGGCTGGAGA	TCTGGACCCATTCCTTCTTG
Cd68	GGGGCTCTTGGGAACTACAC	GTACCGTCACAACCTCCCTG
Tnfα	CCCTCACACTCAGATCATCTTCT	GCTACGACGTGGGCTACAG
Cd36	GGCCAAGCTATTGCGACAT	CAGATCCGAACACAGCGTAGA
Fatp1	CCATCTGGGAGGAGTTCACG	ACACATGCGTGAGGATACGG
Fatp4	CGTTTCGACGGGTACCTCAA	ACATTCTCCCCTTTCCAGCG
Acsl1	CTTTTCTGATTCTGCTGCGGTG	GGGTGTTGGTTGGAAGGGT
Acsl4	CTTCCTCTTAAGGCCGGGAC	TGCCATAGCGTTTTTAGATTTCTTC
U36b4	CGTCCTCGTTGGAGTGACA	CGGTGCGTCAGGGATTG
CycloA	TGGCAAGACCAGCAAGAA	CTCCTGAGCTACAGAAGGAATG

Supplemental Table 3: Primers used in Supplemental Figure 5c

Primer	Forward	Reverse
Angptl4	TTCTGCACCAGAGCAAGTCTAAGTCT	CATGCCTAGGTACTTGCCTAAGT
